# Supplementary material for: The genome sequence of the Eastern Rock Grayling, Hipparchia syriaca (Staudinger, 1871) (Lepidoptera: Nymphalidae)
Source: Wellcome Open Res. 2026 Feb 9;11:98. [Version 1] doi: 10.12688/wellcomeopenres.25847.1 (PMC13033134; doi:10.12688/wellcomeopenres.25847.1)
Supplement: Peer review history

# Reviewer report: Reviewer response for version 1

**DOI:** 10.21956/wellcomeopenres.28448.r150670

**Published:** 2026-03-28

**Authors:** Marco Gerdol

**Affiliations:** 1 University of Trieste, Trieste, Italy

This manuscript provides a report about the genome assembly of *Hipparchia syriaca,*

 From a methodological point of view, it follows a gold standard pipeline, so there is no major concern about the technical soundness of this work.

 The assembly obtained by the authors is of high quality, it reaches a chromosome scale and is characterized by good completeness, even though the alternative haplotype was assembled at a scaffold level.

 Overall, this work does not require revisions and can be accepted for indexing, in my opinion, in its present form.

Are sufficient details of methods and materials provided to allow replication by others?

Yes

Is the rationale for creating the dataset(s) clearly described?

Yes

Are the datasets clearly presented in a useable and accessible format?

Yes

Are the protocols appropriate and is the work technically sound?

Yes

Reviewer Expertise:

Comparative genomics

I confirm that I have read this submission and believe that I have an appropriate level of expertise to confirm that it is of an acceptable scientific standard.

# Reviewer report: Reviewer response for version 1

**DOI:** 10.21956/wellcomeopenres.28448.r150220

**Published:** 2026-03-28

**Authors:** Elena Pazhenkova

**Affiliations:** 1 University of Ljubljana, Ljubljana, Slovenia

The article presents a comprehensive genome assembly of the Eastern Rock Grayling, *Hipparchia syriaca* (Lepidoptera, Nymphalidae), generated as part of the Tree of Life and Project Psyche initiatives. The authors produced a high-quality assembly from a female specimen using PacBio HiFi long-read sequencing combined with Hi-C data for scaffolding and phasing. The resulting assembly comprises two haplotypes with total lengths of 393.56 megabases and 365.79 megabases. 99.87% of haplotype 1 is scaffolded into 30 chromosomal pseudomolecules, including both W and Z sex chromosomes, while haplotype 2 remains at scaffold level. The mitochondrial genome was also assembled, with a length of 15.47 kilobases.

 GenomeScope analysis estimated a haploid genome size of 382.10 megabases, with heterozygosity of 1.67% and repeat content of 28.14%, providing a solid framework for assembly expectations. The sequencing effort yielded approximately 29× coverage of the nuclear genome, complemented by extensive Hi-C data that enabled chromosome-level scaffolding and careful manual curation.

 Importantly, the study incorporates chromosome painting using Merian elements, representing the 32 ancestral linkage groups of Lepidoptera. The distribution of these elements across the assembled chromosomes provides insight into chromosomal organisation and evolutionary rearrangements in *H. syriaca*. This adds an evolutionary genomics perspective to the assembly, enabling comparisons with other lepidopteran genomes and contributing to a broader understanding of chromosome evolution within the group. 

 Overall, this chromosomal-level genome assembly represents a valuable genomic resource for *H. syriaca*. It provides an important foundation for future studies on population genomics, adaptation, and chromosomal evolution in Satyrinae butterflies, particularly given the species’ broad distribution and potential sympatry with closely related taxa. The combination of robust sequencing depth, high assembly quality, and thorough curation makes this work a significant contribution to Lepidoptera genomics and biodiversity research.

Are sufficient details of methods and materials provided to allow replication by others?

Yes

Is the rationale for creating the dataset(s) clearly described?

Yes

Are the datasets clearly presented in a useable and accessible format?

Yes

Are the protocols appropriate and is the work technically sound?

Yes

Reviewer Expertise:

Entomology, bioinformatics, population genomics

I confirm that I have read this submission and believe that I have an appropriate level of expertise to confirm that it is of an acceptable scientific standard.
